# GCLDNet: Gastric cancer lesion detection network combining level feature aggregation and attention feature fusion

**DOI:** 10.3389/fonc.2022.901475

**Published:** 2022-08-29

**Authors:** Xu Shi, Long Wang, Yu Li, Jian Wu, Hong Huang

**Affiliations:** ^1^ Key Laboratory of Optoelectronic Technology and Systems of the Education Ministry of China, Chongqing University, Chongqing, China; ^2^ Department of Pathology, Chongqing University Cancer Hospital and Chongqing Cancer Institute and Chongqing Cancer Hospital, Chongqing, China; ^3^ Head and Neck Cancer Center, Chongqing University Cancer Hospital and Chongqing Cancer Institute and Chongqing Cancer Hospital, Chongqing, China

**Keywords:** artificial intelligence, deep learning, image segmentation, convolutional neural network, gastric cancer lesion detection, level feature aggregation, attention feature fusion

## Abstract

**Background:**

Analysis of histopathological slices of gastric cancer is the gold standard for diagnosing gastric cancer, while manual identification is time-consuming and highly relies on the experience of pathologists. Artificial intelligence methods, particularly deep learning, can assist pathologists in finding cancerous tissues and realizing automated detection. However, due to the variety of shapes and sizes of gastric cancer lesions, as well as many interfering factors, GCHIs have a high level of complexity and difficulty in accurately finding the lesion region. Traditional deep learning methods cannot effectively extract discriminative features because of their simple decoding method so they cannot detect lesions accurately, and there is less research dedicated to detecting gastric cancer lesions.

**Methods:**

We propose a gastric cancer lesion detection network (GCLDNet). At first, GCLDNet designs a level feature aggregation structure in decoder, which can effectively fuse deep and shallow features of GCHIs. Second, an attention feature fusion module is introduced to accurately locate the lesion area, which merges attention features of different scales and obtains rich discriminative information focusing on lesion. Finally, focal Tversky loss (FTL) is employed as a loss function to depress false-negative predictions and mine difficult samples.

**Results:**

Experimental results on two GCHI datasets of SEED and BOT show that DSCs of the GCLDNet are 0.8265 and 0.8991, ACCs are 0.8827 and 0.8949, JIs are 0.7092 and 0.8182, and PREs are 0.7820 and 0.8763, respectively.

**Conclusions:**

Experimental results demonstrate the effectiveness of GCLDNet in the detection of gastric cancer lesions. Compared with other state-of-the-art (SOTA) detection methods, the GCLDNet obtains a more satisfactory performance. This research can provide good auxiliary support for pathologists in clinical diagnosis.

## 1 Introduction

Gastric cancer is a type of cancer caused by the immortal proliferation of abnormal cells in the stomach, and it is the fifth most common type of cancer all over the world (1, 2), which seriously affects people’s health. Gastric cancer has a high morbidity and mortality rate and is the world’s third largest disease related to cancer deaths (3). The specific survival period of gastric cancer is 12 months, and 90% of patients will die within 5 years (4). It is one of the most aggressive and most deadly cancer types (5); thus, accurate diagnosis of gastric cancer as early as possible is extremely important.

In actual clinical practice, methods such as endoscopy and imaging examination can detect abnormalities in the stomach. However, whether there is gastric cancer can only be diagnosed through histopathological examination, and histopathological diagnosis is the gold standard for clinical medicine diagnosis (6, 7). Therefore, it is significant to perform a biopsy on the patient’s stomach; biopsy refers to removing epithelial tissue from the patient to make a section for further examination during gastroscopy. However, histopathological images (HIs) are characterized by a large amount of data, and pathologists have a large amount of manual identification work, and continuous work for a long time will also affect the reliability of results. At the same time, pathologists in this field are scarce, and it is essential to find new ways to address these issues (8). With the rapid development of computer vision technology (9), the utilization of artificial intelligence (AI) methods, especially deep learning, can not only omit the time-consuming procedure where doctors search for cancerous tissues, but also improve the accuracy of diagnosis (10), and this is beneficial to realizing the automated and accurate detection of gastric cancer.

The good performance of deep learning has brought great opportunities to medical image analysis. Therefore, various methods based on deep learning are designed in HI segmentation. Xiao et al. (11) proposed a polar representation-based algorithm for non-small lung cancer segmentation from HIs. In cell nucleus segmentation, Pan et al. (12) developed an algorithm combining sparse reconstruction and deep convolution network to overcome the variability and complexity of size, shape, and texture of breast cancer cell nucleus. In liver cancer cell nucleus segmentation, Shyam et al. (13) designed a NucleiSegNet, which includes a residual block, a bottleneck block, and an attention module. Anirudh et al. (14) presented an encoder–decoder network combined with atrous spatial pyramid pooling and attention module for kidney cell nucleus segmentation. In prostate cancer detection, Massimo et al. (15) adopted a hybrid segmentation strategy based on gland contour structure and deep learning. Li et al. (16) applied an EM-based semi-supervised deep learning method for prostate HI segmentation on limited annotated datasets. In breast cancer HI segmentation, David et al. (17) proposed a deep multi-magnification network to extract spatial features within a class and learn spatial relationships among classes. Blanca et al. (18) designed an encoder–decoder network combining separable atrous convolution and conditional random field. In addition, Meng et al. (19) introduced a triple upsampling segmentation network with distribution consistency loss for HI lesion diagnosis and achieved excellent performance on three HI datasets of cervical cancer, colon cancer, and liver cancer. Amit et al. (20) introduced a separable convolution pyramid pooling network and achieved good performance on kidney and breast HIs. These research works in the above various HIs show that deep learning can improve the detection efficiency of some diseases, and this will reduce the workload of pathologists.

In recent years, deep learning algorithms have been widely explored to perform classification and segmentation in GCHIs (21). Qu et al. (22) proposed an improved deep learning algorithm for GCHI classification based on step-by-step fine-tuning and alleviating data shortage problems by establishing an intermediate dataset. Harshita et al. (23) employed the AlexNet deep convolutional networks to extract feature information of GCHIs for classification, and final overall accuracy is 69.90%. Osamu et al. (24) combined convolutional neural networks (CNNs) and recurrent neural networks (RNNs) to classify gastric HIs; the AUC value of model reached 97.00% and 99.00% for gastric adenocarcinoma and adenoma, respectively. However, the classification of GCHIs is of limited significance, because not all regions in images are cancer tissues. At the same time, pixel-based segmentation can achieve more accurate detection of gastric cancer lesions; thus, many studies focus on segmentation methods. To solve the problems of complexity and small morphological difference between diseased cells and normal cells of GCHIs, Chen et al. (6) designed an ADEU-Net, which uses transfer learning model to enhance feature extraction ability, and short connection is employed to promote fusion of deep and shallow features. Aghababaie et al. (25) developed a V-Net (a fully convolution neural network) for normal and diseased tissue segmentation from GCHIs, Dice coefficient reached 96.18%, and Jac index reached 92.77%. Furthermore, obtaining and utilizing the multi-scale features of GCHIs is key to getting satisfactory segmentation performance in gastric cancer detection. Sun et al. (26) introduced deformed convolution and multi-scale embedding networks for GCHI segmentation, which used spatial pyramid modules and encoding–decoding-based embedding networks to achieve multi-scale segmentation. Accuracy reached 91.60%, and IoU coefficient reached 82.65%. Li et al. (27) proposed a GastricNet network, which employed different structures to deep and shallow layers for better feature extraction. Qin et al. (10) presented a detection algorithm based on the feature pyramid structure, which can focus on global context information from high-level features. Although the aforementioned methods have achieved impressive results, most of these studies only use existing algorithms in natural image detection. These studies do not consider the effective fusion of deep and shallow features in the decoding stage, resulting in unsatisfactory detection results.

**Figure 1:**
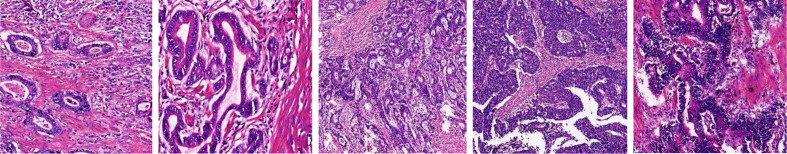
Example images demonstrating the complexity of GCHIs lesions.

The problems from previous work can be summarized as follows: (1) The characteristics of GCHIs themselves: as shown in **Figure 1**, GCHIs have the problems of high complexity and difficulty in localization of gastric cancer lesions. The specific manifestations include different sizes of gastric cancer lesions, large differences in the morphology of different lesions, and many surrounding interference factors, among others. (2) Research on detection algorithms is not targeted enough: classic segmentation network is not necessarily suitable for GCHIs, because the traditional segmentation network is mainly used in natural images, and few networks are dedicatedly designed for the characteristics of GCHIs, resulting in traditional deep learning methods that cannot effectively extract deep features and detect gastric cancer lesions. (3) Many works do not consider the effective fusion of deep and shallow features and the extraction of discriminative features, resulting in limited segmentation performance.

To solve the above-mentioned problems, the GCLDNet is proposed. The main contributions of the GCLDNet in detecting gastric cancer lesions are as follows.

1. The GCLDNet designs a level feature aggregation (LFA) structure in the decoding stage, which aggregates rich feature information in the last layer under different receptive fields. It can make the most out of the deep and shallow feature information to effectively extract deeper features of GCHIs.
2. An attention feature fusion module (AFFM) is proposed to accurately locate the gastric cancer lesion area. It merges attention features of different scales, and finally obtains rich discriminative feature information focusing on the gastric cancer lesion area.

The rest of this paper is arranged as follows. *Section 2* mainly introduces the two datasets used in this paper and presents the proposed GCLDNet method in detail. To evaluate the performance of the GCLDNet method, *Section 3* discusses the experimental results on SEED and BOT datasets. Finally, *Section 4* concludes this paper and gives some suggestions for our future work.

## 2 Materials and methods

### 2.1 Materials

#### 2.1.1 Two datasets

**Figure 2:**
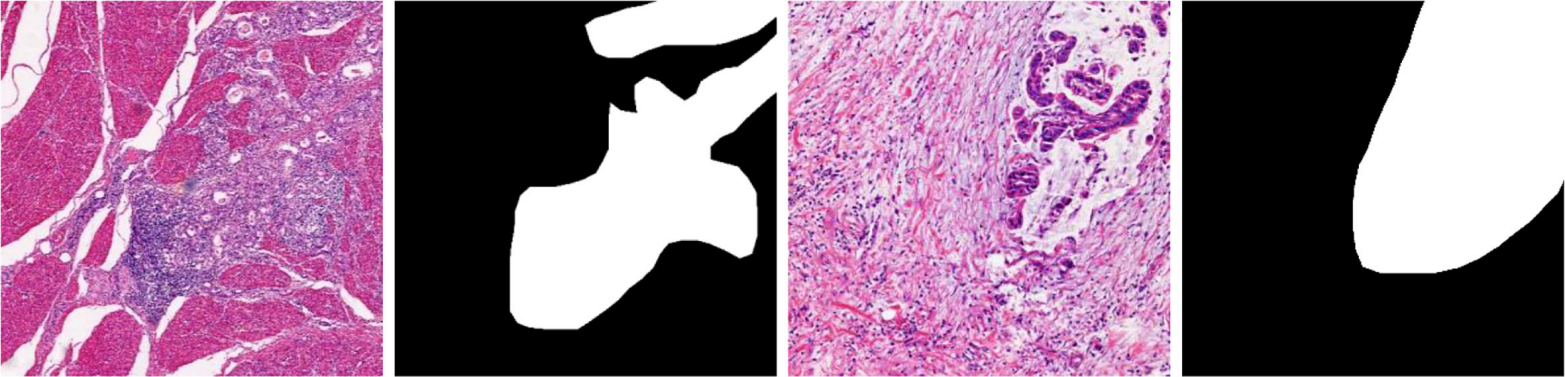
Example images and masks of the SEED dataset.

**Figure 3:**
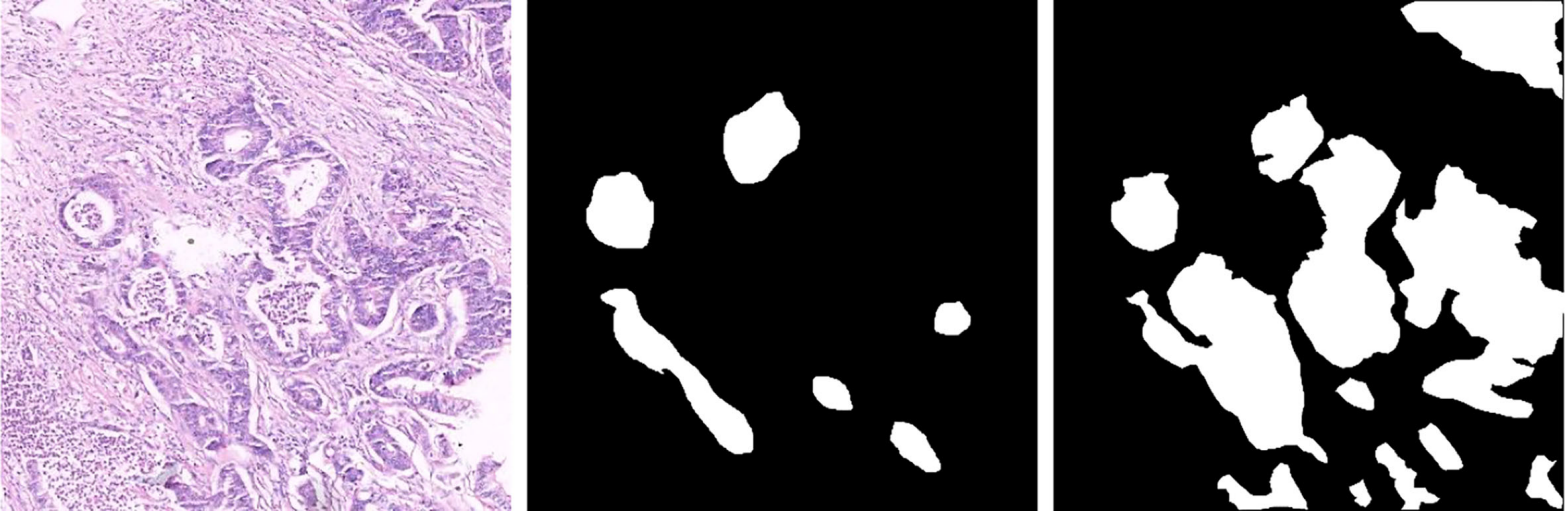
Gastric cancer histopathological image, original annotated mask image, and supplementary annotated mask image of the BOT dataset.

- SEED dataset: This dataset comes from The Second Jiangsu Big Data Development and Application Competition (Medical and Health Track, https://www.jseedata.com). It contains 574 normal images, 1,196 images with gastric cancer lesions, and the corresponding annotated mask images. The format of both image and mask is.png, and images are all collected under a 20× magnification field of view. Example images are shown in **Figure 2**.
- BOT dataset: This dataset comes from The 2017 China Big Data Artificial Intelligence Innovation and Entrepreneurship Competition (Pathology Slice Recognition AI Challenge, https://data.mendeley.com/datasets/thgf23xgy7). It contains 560 HIs of gastric cancer and the corresponding annotated mask images. All images are collected under a 20× magnification field of view. These slices are stained with hematoxylin–eosin (H&E) by the anatomic pathologist. An example image is displayed in **Figure 3**. It is worth noticing that only part of the lesion area in the GT image of the BOT dataset is annotated (28). This will have a serious impact on the training of the model. To address this problem, we invited relevant experienced pathologists to fully annotate this dataset.

#### 2.1.2 Data preprocessing

In data preprocessing, data augmentations are employed to improve the performance of image segmentation, including sample centering, horizontal flipping, and vertical flipping. The sample centering operation refers to subtracting the sample mean so that the mean of the new sample is zero. The horizontal flipping and vertical flipping operations refer to flipping image horizontally and vertically, respectively. Two datasets are randomly divided into a training set and a test set at a ratio of 8:2. To preserve image details as much as possible while reducing the number of parameters of the model, the image sizes of the two datasets are uniformly resized to 512 × 512 during training.

#### 2.1.3 Experimental software and hardware environment

In the experiment, the hardware environment is based on the PANYAO 7048GR server, the memory is 256G, and the graphics card is NVIDIA TITAN RTX (Memory is 24G, Tensor Cores is 576, CUDA Cores is 4608). To implement our proposed GCLDNet method, the deep learning framework Tensorflow 2.2 is employed, and the programming language is Python 3.8.

### 2.2 Methods

**Figure 4:**
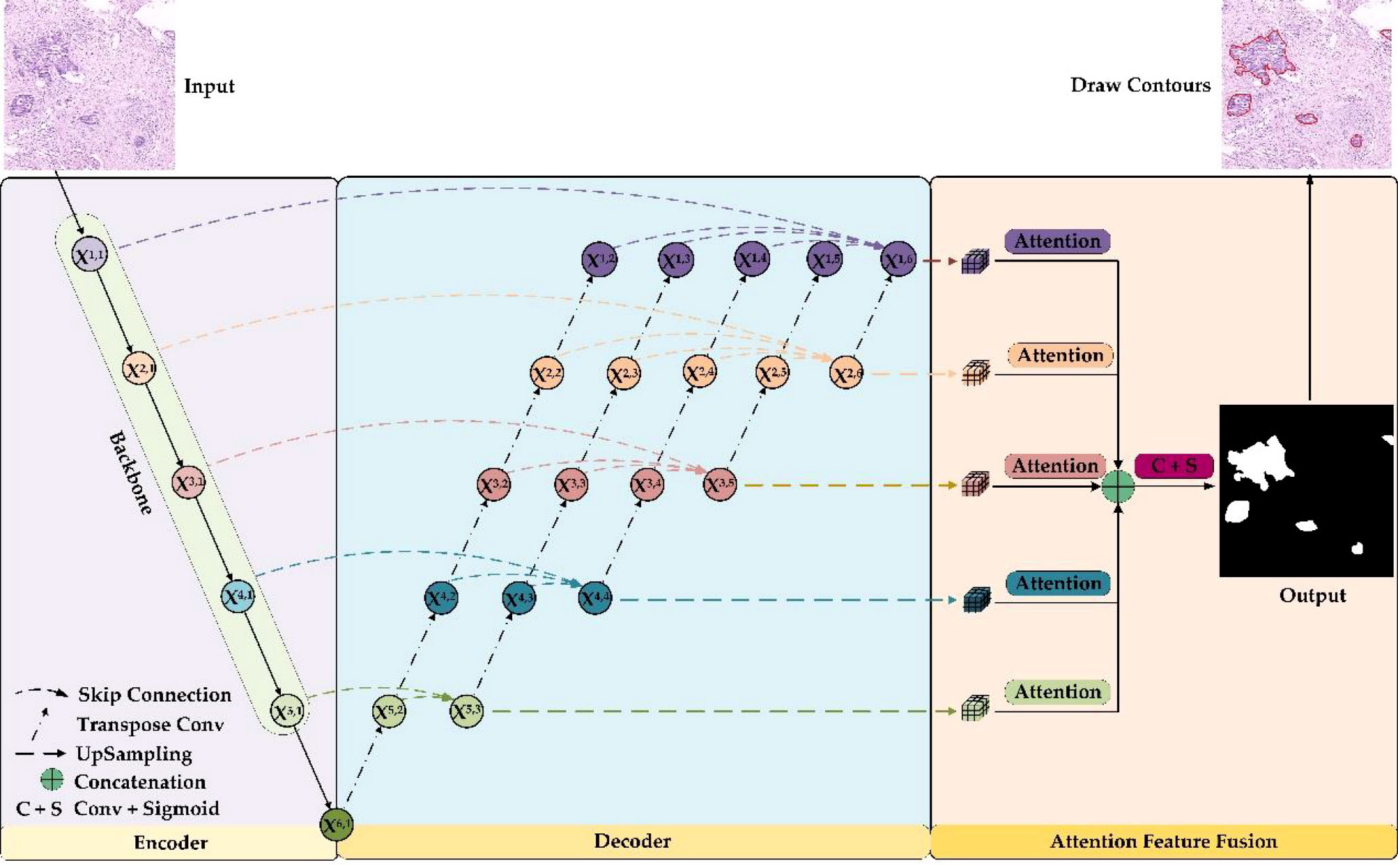
The overall framework of the GCLDNet.

The overall framework of the GCLDNet is presented in **Figure 4**. It includes LFA and AFFM. The GCLDNet aggregates shallow and deep features through skip connections then adopts the AFFM to better extract discriminative features in GCHIs and merges deep features at different scales to improve segmentation accuracy.

#### 2.2.1 Level feature aggregation

In GCHIs, there are large variances in the shape and size of the lesion area, as well as many surrounding interfering factors. The general U-Net network cannot effectively extract deep features, or cannot extract some discriminative features due to its simple decoder structure (29). To alleviate these problems, we employ multiple skip connections to form a feature aggregation structure. Through aggregating features among various levels, the GCLDNet can extract in-depth features from GCHIs, to make the segmentation results of model better.

As can be seen from **Figure 4**, the GCLDNet redesigns the decoder structure by using multiple skip connections. In this paper, we assume that *X* ^i,j^ is the output feature map of the *j*th position of the *i*th layer (the possible values of *i* and *j* are both {1,2,…,6}), and it should be noted that *X* ^i,1^ is the output feature map at each stage of the encoder. The overall idea of the decoder structure is to perform feature aggregation from bottom to top and from left to right. First, for the bottom feature *X* ^6,1^, perform five times consecutive transposed convolutions on it to obtain feature maps *X* ^5,2^, *X* ^4,2^, *X* ^3,2^, *X* ^2,2^, and *X* ^1,2^, respectively (the upsampling rate is 2). Then, the feature maps *X* ^5,1^ and *X* ^5,2^ are aggregated by skip connections in the direction from left to right to obtain the depth feature *X* ^5,3^. Finally, the depth aggregation features *X* ^4,4^, *X* ^3,5^, *X* ^2,6^, and *X* ^1,6^ under the remaining receptive fields are also obtained through similar operations. In this way, the depth features obtained by aggregating the deep and shallow features contain richer semantic information, which is helpful for the identification of gastric cancer lesions. This is the general idea of designing the decoder, and the details of implementation will be introduced next.

In the following, we will explain feature aggregation at each resolution. Taking *X* ^1,i^ (i = 1,2,…,6) layer features as an instance, the initial feature is *X* ^1,1^, and the final aggregated feature map is *X* ^1,6^. *X* ^1,1^ is the first feature map to be aggregated. The second feature map to be aggregated is *X* ^1,2^, which is obtained after five times consecutive upsampling operations to *X* ^6,1^. The expression to get feature maps *X* ^1,2^ can be computed as follows:


(1) $$X^{1,2}=F_{\text{U}}^{1}(X^{2,2})=F_{\text{U}}^{2}(X^{3,2})=F_{\text{U}}^{3}(X^{4,2})=F_{\text{U}}^{4}(X^{5,2})=F_{\text{U}}^{5}(X^{6,1})$$

where $F_{U}^{\text{i}}$ (•) means that the number of consecutive upsampling operations (transposed convolution) is i. The third feature map to be aggregated is *X* ^1,3^, which is gained by *X* ^5,3^ after four times consecutive upsampling operations. It can be denoted as


(2) $$X^{1,3}=F_{\text{U}}^{1}(X^{2,3})=F_{\text{U}}^{2}(X^{3,3})=F_{\text{U}}^{3}(X^{4,3})=F_{\text{U}}^{4}(X^{5,3})$$

For the remaining features to be aggregated, *X* ^1,4^, *X* ^1,5^, and $X_{\text{U}}^{1,6}$ are obtained to the same size by the same consecutive upsampling methods from *X* ^4,4^, *X* ^3,5^, and *X* ^2,6^. This series of operations can be expressed as the following:


(3) $$X^{1,4}=F_{\text{U}}^{1}(X^{2,4})=F_{\text{U}}^{2}(X^{3,4})=F_{\text{U}}^{3}(X^{4,4})$$

(4) $$X^{1,5}=F_{\text{U}}^{1}(X^{2,5})=F_{\text{U}}^{2}(X^{3,5})$$

(5) $$X_{\text{U}}^{1,6}=F_{\text{U}}^{1}(X^{2,6})$$

Finally, the aggregated feature *X* ^1,6^ is presented as


(6) $$X^{1,6}=X^{1,1}⊕X^{1,2}⊕X^{1,3}⊕X^{1,4}⊕X^{1,5}⊕X_{\text{U}}^{1,6}$$

Equation (6) is the expression of the final aggregated features of first level. The expressions of the remaining layers can be summarized as follows:


(7) $$X{\;}^{\text{i,j}}=\sum_{\text{i}}⊕X^{\text{ i,k}}i=2,3,4,5;j=8-i;k=1,2,\ldots ,j-1.$$

where ∑⊕ represents continuous concatenation operation. Then, the feature aggregation of each layer is completed.

#### 2.2.2 Attention feature fusion module

**Figure 5:**
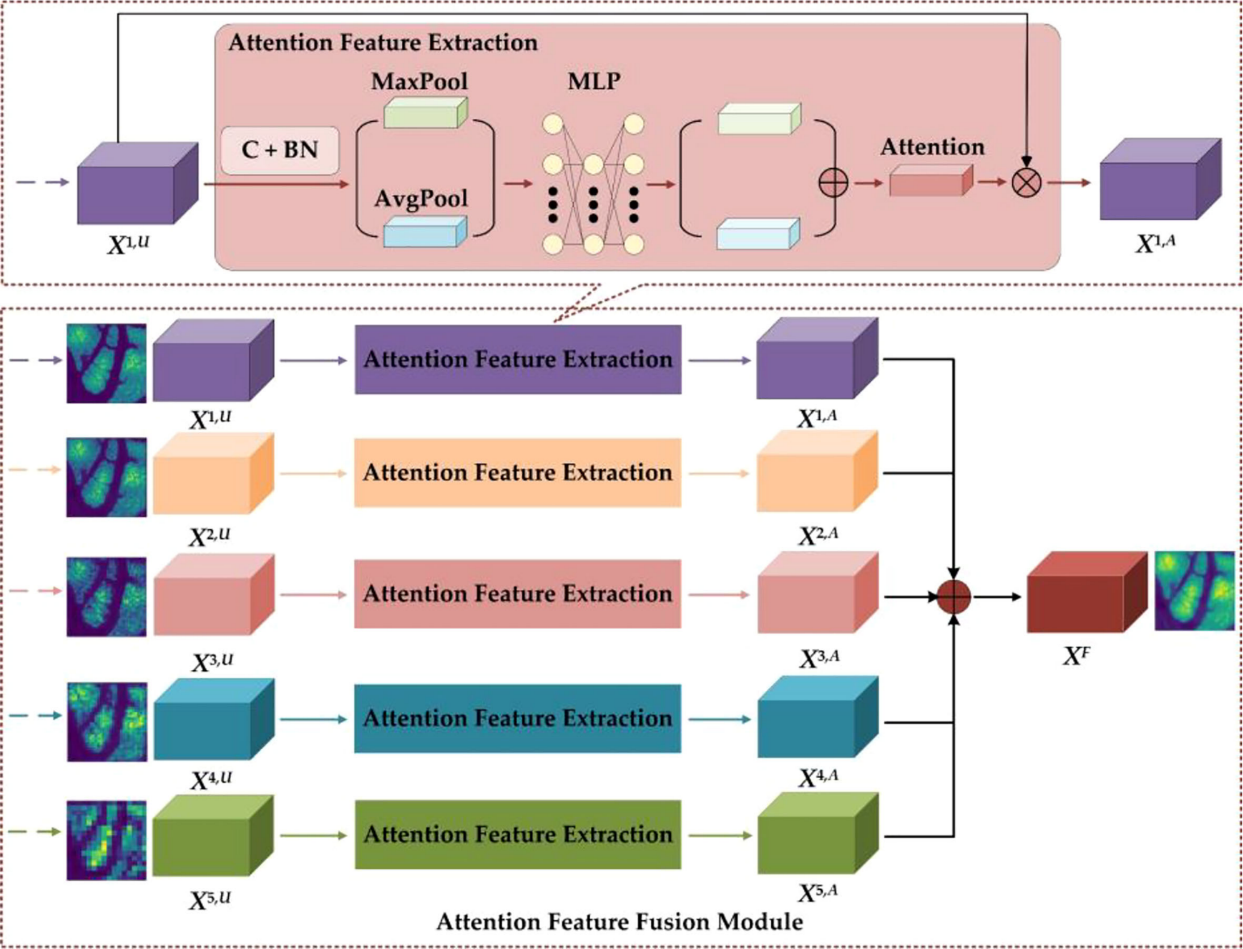
Details of the AFFM.

The details of the AFFM are shown in **Figure 5**. To restore feature map size to the original GCHI size, the GCLDNet first performs an upsampling operation on features of different scales (the specific method is bilinear interpolation). In AFFM, the GCLDNet utilizes the attention mechanism (29, 30) to make extracted features pay more attention to the gastric cancer lesion area so that the extracted features are more discriminative. Then, features of different scales are fused to make good use of feature information under different receptive fields to improve segmentation accuracy of GCHIs.

Taking the *X* ^1^ layer feature as an example, the feature map obtained by upsampling operation to *X* ^1,6^ is *X* ^1,U^. To enhance the expression of features, the GCLDNet first applies a 3×3 convolution operation on *X* ^1,U^. Assuming that the obtained feature is *X*_C_, it can be computed as


(8) XC=f(∑j=1CXj 1,U*W+b)

where *W* and *b* are the weight parameters that can be learned, *f* (•) is the activation function, and * indicates convolution operation. Moreover, each convolution layer on the feature map are followed by batch normalization (BN) operation, which can speed up the convergence speed and increase the stability of model. Assuming that the feature obtained through the BN layer is X_B_, it can be calculated *via*


(9) $$X_{\text{B}}=\gamma \frac{X_{\text{C}}-E\left(X_{\text{C}}\right)}{\sqrt{Var\left(X_{\text{C}}\right)}}+\beta$$

where *E*(•) and *Var*(•) represent the mathematical expectation and variance functions, respectively, and *γ* and *β* are learnable parameters in the BN layer of the network. To fully extract the attention weight features, the GCLDNet uses global average pooling and global maximum pooling operations. Assuming that the obtained attention weights are *P*_Avg_ and *P*_Max,_ respectively, they can be formulated as


(10) $$P_{Avg}\text{ = }\frac{1}{\text{H}\times \text{W}}\sum_{i=1}^{\text{H}}\sum_{j=1}^{\text{W}}X_{\text{B}}\text{(i,j)}$$

(11) $$P_{\text{Max}}=Max(X_{\text{B}})$$

where *P*_Avg_∈R^C×1×1^, *P*_Max_∈R^C×1×1^. To further extract more in-depth discriminative features, the GCLDNet uses a multi-layer perception (MLP) with a hidden layer and shared weights. At the same time, considering the computational cost, the number of neurons in the middle layer is reduced to C/r, where r is the reduction rate. The weights of two groups are added according to the corresponding elements, and finally the sigmoid activation function σ is adopted to obtain the attention weight coefficient, denoted as *W*_A_. The above process can be given as the following:


(12) $$W_{\text{A}}=\sigma (MLP(P_{\text{Avg}})+MLP(P_{\text{Max}}))$$

It is worth noticing that *W*_A_∈R^C×1×1^ and the value range of *W*_A_ is [0,1].

After that, the weight *W*_A_ and the input feature map *X* ^1,U^ are correspondingly multiplied to obtain the attention feature map, denoted as *X* ^1,A^; it can be expressed as


(13) $$X^{1,A}=W_{\text{A}}⊗X^{1,\text{U}}$$

Lastly, the features obtained under different receptive fields are concatenated (31). The final feature is *X* ^F^, and the feature fusion strategy can be represented by


(14) $$X^{\text{F}}=\sum_{\text{i}}⊕X{\;}^{\text{i,A}}i=1,2,\ldots ,5$$

where *X* ^F^ is the final output feature of AFFM.

#### 2.2.3 Focal Tversky loss function

In deep learning, the loss function measures the predictive ability of the model. Loss function is minimized to make the model reach a stable state of convergence. At the same time, the error between the predicted value and the true value is the smallest.

In the field of medical image segmentation, commonly used loss functions include cross entropy loss function (BCE-Loss) and dice loss function (Dice-Loss). However, BCE-Loss is susceptible to category imbalance. To evaluate the similarity between the gastric cancer area predicted by the model and the real area, the Dice similarity coefficient is often used as evaluation index; hence, the loss function Dice-Loss can be designed according to Dice (32). However, Dice-Loss has equal predictive weights for false positives and false negatives. This is an obvious limitation such that the impairment of false negatives is greater than false positives in actual medical applications. Predicting the tissue in the gastric cancer area as normal tissue will have a serious impact on the doctor’s diagnosis and cause patients to miss the best treatment time. At the same time, the small gastric cancer area does not contribute much to loss function; thus, loss function is not sensitive to it during the training process. To address the above challenges, the GCLDNet uses focal Tversky loss (FTL) (33) as a loss function to reduce false-negative predictions and mine difficult samples in GCHIs. It can be defined as


(15) $$L_{FTL}={\left(1-\frac{\left|Y^{\text{GT}}\cap {\text{Y}}^{\text{Pre}}\right|}{\left|Y^{\text{GT}}\cap Y^{\text{Pre}}\right|\;+\;\alpha \;\left|Y^{\text{GT}}\;-\;Y^{\text{Pre}}\right|\;+\text{ β }\left|Y^{\text{Pre}}\;-\;Y{\;}^{\text{GT}}\right|}\right)}^{\frac{1}{\lambda }}$$

where *α* and *β* are balance parameters. By adjusting *α* and *β*, fewer false-negative predictions can be obtained. *λ* is adopted to increase the contribution of a small gastric cancer area to the loss function. Generally speaking, the suitable value range of *λ* is [1,3]. *Y* ^GT^ represents the GT image, and *Y* ^Pre^ is the corresponding predicted image. |•| indicates the number of a set. |*Y* ^GT^∩*Y* ^Pre^| represents the overlap between the predicted area and the ground truth area (true positive, TP). |*Y* ^GT^-*Y* ^Pre^| measures the number of misclassified gastric cancer tissue to the background (false negative, FN). |*Y* ^Pre^-*Y* ^GT^| denotes the region that misclassified the background to gastric cancer tissue (false positive, FP). |*Y* ^GT^-*Y* ^Pre^|+|*Y* ^Pre^-*Y* ^GT^| represents the number of all misclassified pixels.

In gastric cancer image detection, there are two categories, namely, gastric cancer tissue and background. Therefore, the above equation can be transformed into the following calculation equation:


(16) $$L_{FTL}={\left(1-\frac{\sum_{i=1}^{N}p_{1i}g_{1i}}{\sum_{i=1}^{N}p_{1i}g_{1i}+\alpha \sum_{i=1}^{N}p_{1i}g_{0i}+\beta \sum_{i=1}^{N}p_{0i}g_{1i}}\right)}^{\frac{1}{\lambda }}$$

where *N* indicates the total number of pixels,  p_0i_  and  p_1i_  respectively represent the probability that the *i*th pixel is predicted to be the background and gastric cancer.  g_0i_  means that the value is 1 when the *i*th pixel is the background; otherwise, it is 0.  g_1i_  indicates that the value is 1 when the *i*th pixel is gastric cancer; otherwise, it is 0.

The gradient of the loss in Equation (16) with respect to  p_1i_  and  p_0i_  can be calculated as


(17) $$\begin{array}{c} \frac{\partial L_{FTL}}{\partial p_{1i}}=\frac{1}{\lambda }{\left(1-\frac{\sum_{i=1}^{N}p_{1i}g_{1i}}{\sum_{i=1}^{N}p_{1i}g_{1i}+\alpha \sum_{i=1}^{N}p_{1i}g_{0i}+\beta \sum_{i=1}^{N}p_{0i}g_{1i}}\right)}^{\frac{1}{\lambda }-1}\frac{g_{1i}\left(\sum_{i=1}^{N}p_{1i}g_{1i}+\alpha \sum_{i=1}^{N}p_{1i}g_{0i}+\beta \sum_{i=1}^{N}p_{0i}g_{1i}\right)-\left(g_{1i}+\alpha g_{0i}\right)\left(\sum_{i=1}^{N}p_{1i}g_{1i}\right)}{{\left(\sum_{i=1}^{N}p_{1i}g_{1i}+\alpha \sum_{i=1}^{N}p_{1i}g_{0i}+\beta \sum_{i=1}^{N}p_{0i}g_{1i}\right)}^{2}}\\ =\frac{1}{\lambda }{\left(1-\frac{\sum_{i=1}^{\text{N}}{\text{p}}_{1i}{\text{g}}_{1i}}{\sum_{i=1}^{\text{N}}{\text{p}}_{1i}{\text{g}}_{1i}+\alpha \sum_{i=1}^{\text{N}}{\text{p}}_{1i}{\text{g}}_{0i}+\beta \sum_{i=1}^{\text{N}}{\text{p}}_{0i}{\text{g}}_{1i}}\right)}^{\frac{1}{\lambda }}\frac{\sum_{i=1}^{\text{N}}{\text{p}}_{1i}{\text{g}}_{1i}+\alpha \sum_{i=1}^{N}p_{1i}g_{0i}+\beta \sum_{i=1}^{N}p_{0i}g_{1i}}{\alpha \sum_{i=1}^{N}p_{1i}g_{0i}+\beta \sum_{i=1}^{N}p_{0i}g_{1i}}\frac{g_{1i}\left(\sum_{i=1}^{N}p_{1i}g_{1i}+\alpha \sum_{i=1}^{N}p_{1i}g_{0i}+\beta \sum_{i=1}^{N}p_{0i}g_{1i}\right)-\left(g_{1i}+\alpha g_{0i}\right)\left(\sum_{i=1}^{N}p_{1i}g_{1i}\right)}{{\left(\sum_{i=1}^{N}p_{1i}g_{1i}+\alpha \sum_{i=1}^{N}p_{1i}g_{0i}+\beta \sum_{i=1}^{N}p_{0i}g_{1i}\right)}^{2}}\\ =\frac{1}{\lambda }{\left(1-\frac{\sum_{i=1}^{N}p_{1i}g_{1i}}{\sum_{i=1}^{N}p_{1i}g_{1i}+\alpha \sum_{i=1}^{N}p_{1i}g_{0i}+\beta \sum_{i=1}^{N}p_{0i}g_{1i}}\right)}^{\frac{1}{\lambda }}\frac{g_{1i}\left(\sum_{i=1}^{N}p_{1i}g_{1i}+\alpha \sum_{i=1}^{N}p_{1i}g_{0i}+\beta \sum_{i=1}^{N}p_{0i}g_{1i}\right)-\left(g_{1i}+\alpha g_{0i}\right)\left(\sum_{i=1}^{N}p_{1i}g_{1i}\right)}{\left(\alpha \sum_{i=1}^{N}p_{1i}g_{0i}+\beta \sum_{i=1}^{N}p_{0i}g_{1i}\right)\left(\sum_{i=1}^{N}p_{1i}g_{1i}+\alpha \sum_{i=1}^{N}p_{1i}g_{0i}+\beta \sum_{i=1}^{N}p_{0i}g_{1i}\right)} \end{array}$$

(18) $$\begin{array}{c} \frac{\partial L_{FTL}}{\partial p_{0i}}=\frac{1}{\lambda }{\left(1-\frac{\sum_{i=1}^{N}p_{1i}g_{1i}}{\sum_{i=1}^{N}p_{1i}g_{1i}+\alpha \sum_{i=1}^{N}p_{1i}g_{0i}+\beta \sum_{i=1}^{N}p_{0i}g_{1i}}\right)}^{\frac{1}{\lambda }-1}\frac{-\beta g_{1i}\left(\sum_{i=1}^{N}p_{1i}g_{1i}\right)}{{\left(\sum_{i=1}^{N}p_{1i}g_{1i}+\alpha \sum_{i=1}^{N}p_{1i}g_{0i}+\beta \sum_{i=1}^{N}p_{0i}g_{1i}\right)}^{2}}\\ =\frac{1}{\lambda }{\left(1-\frac{\sum_{i=1}^{N}p_{1i}g_{1i}}{\sum_{i=1}^{N}p_{1i}g_{1i}+\alpha \sum_{i=1}^{N}p_{1i}g_{0i}+\beta \sum_{i=1}^{N}p_{0i}g_{1i}}\right)}^{\frac{1}{\lambda }}\frac{\sum_{i=1}^{N}p_{1i}g_{1i}+\alpha \sum_{i=1}^{N}p_{1i}g_{0i}+\beta \sum_{i=1}^{N}p_{0i}g_{1i}}{\alpha \sum_{i=1}^{N}p_{1i}g_{0i}+\beta \sum_{i=1}^{N}p_{0i}g_{1i}}\frac{-\beta g_{1i}\left(\sum_{i=1}^{N}p_{1i}g_{1i}\right)}{{\left(\sum_{i=1}^{N}p_{1i}g_{1i}+\alpha \sum_{i=1}^{N}p_{1i}g_{0i}+\beta \sum_{i=1}^{N}p_{0i}g_{1i}\right)}^{2}}\\ =-\frac{1}{\lambda }{\left(1-\frac{\sum_{i=1}^{N}p_{1i}g_{1i}}{\sum_{i=1}^{N}p_{1i}g_{1i}+\alpha \sum_{i=1}^{N}p_{1i}g_{0i}+\beta \sum_{i=1}^{N}p_{0i}g_{1i}}\right)}^{\frac{1}{\lambda }}\frac{\beta g_{1i}\left(\sum_{i=1}^{N}p_{1i}g_{1i}\right)}{\left(\alpha \sum_{i=1}^{N}p_{1i}g_{0i}+\beta \sum_{i=1}^{N}p_{0i}g_{1i}\right)\left(\sum_{i=1}^{N}p_{1i}g_{1i}+\alpha \sum_{i=1}^{N}p_{1i}g_{0i}+\beta \sum_{i=1}^{N}p_{0i}g_{1i}\right)} \end{array}$$

It is noticed that the FTL is the same as the dice loss when *α* =*β* = 0.5 and *λ* =1.

## 3 Results and discussion

### 3.1 Training process

Stochastic Gradient Descent (SGD) (34) is employed as a model optimizer during training, and prediction results of model at different depths are used for joint collaborative training. In addition, the batch size is set to 6, the total number of training epochs is set to 150 in experiments, and the initial learning rate is 1e-2. During model training, when the dice coefficient does not increase for five consecutive epochs, the learning rate will be halved, and the minimum learning rate is 1e-6. All parameters are optimal values or better values obtained after many experiments. In addition, to ensure the accuracy of experimental results, the average of five experimental results is used as final results.

### 3.2 Evaluation metrics

Four metrics are employed to quantitatively evaluate the performance of the GCLDNet, including Dice similarity coefficient (DSC), Jaccard index (JI), Accuracy (ACC), and Precision (PRE). The specific formulas are as follows:


(19) $$\text{DSC=}\frac{2\left|Y^{\text{GT}}\cap Y^{\text{Pre}}\right|}{\left|Y^{\text{GT}}\right|+\left|Y^{\text{Pre}}\right|}$$

(20) $$\text{JI=}\frac{\left|Y^{\text{GT}}\cap Y^{\text{Pre}}\right|}{\left|Y^{\text{GT}}\cup Y^{\text{Pre}}\right|}$$

(21) $$\text{ACC=}\frac{TP+TN}{TP+FP+TN+FN}$$

(22) $$\text{PRE=}\frac{TP}{TP+FP}$$

where *Y* ^GT^ is the region-of-interest (ROI) pixel point set marked by the expert, *Y* ^Pre^ is the predicted ROI pixel point set, *TN* is true negative, *TP* is true positive, *FN* is false negative, and *FP* is false positive. These four metrics evaluate the effect of model segmentation from four different angles, making the evaluation results more reasonable and objective.

### 3.3 Comparative experiment

In order to prove the effectiveness of the GCLDNet method, we selected some SOTA networks for comparison, including FCN (35), U-Net (36), UNet++ (37), FPN (38), PSPNet (39), SegNet (40), LinkNet (41), DeepLabV3 (42), MultiResUNet (43), CCBANet (44), U2net (45), and UNet3+ (46). To ensure the fairness of comparison process, comparative methods also adopt the same preprocessing method as GCLDNet and the parameters are adjusted to the optimal state.

**Figure 6:**
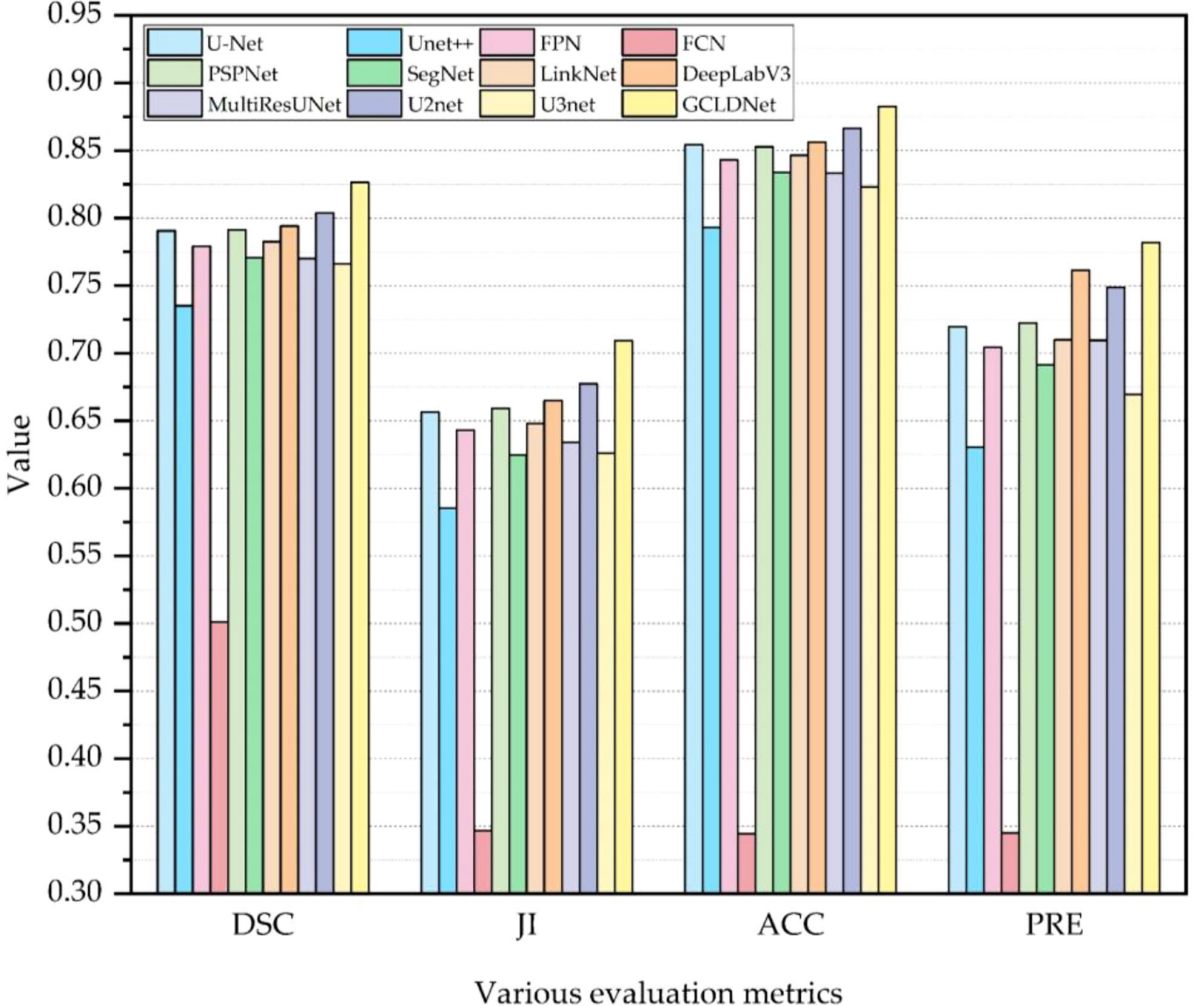
Visual comparison of the performance of different models on the SEED dataset.

The comparison results of different algorithms on the SEED dataset are presented in **Table 1** and **Figure 6**. Furthermore, these methods are separated into two categories, namely, Encoder–Decoder-based methods (†) and Multi-scale-based methods (◊). Experimental results show that our proposed method achieves the best performance on four metrics. Based on the basic Encoder–Decoder structure, SegNet improves the simple decoder structure of FCN so that it achieves relatively good results. However, SegNet has the problem of ineffective integration of deep and shallow features. UNet has made great progress in medical image segmentation, which indicates the importance of skip connections for accuracy improvement. Nevertheless, the decoding processes of UNet++ and UNet3+ fail to make good use of in-depth features from skip concatenations, which results in a poor effect. Notably, U2Net performs an impressive segmentation effect because of its embedded encoding–decoding structure, but the accuracy is inferior to the GCLDNet and the number of parameters also exceeds our proposed method. In multi-scale structure models, the results of PSPNet and DeepLabV3 reveal that extracting certain multi-scale features contributes to segmentation performance. The number of parameters of DeepLabV3 is the highest among these networks, but the segmentation performance does not achieve the best, which demonstrates that the DeepLabV3 fails to use the multi-scale features of lesions adequately. In summary, the proposed GCLDNet performs better than other SOTA methods and the number of parameters are moderate.

**Table 1:** Segmentation performance of different models on the SEED dataset.

Method type	Methods	DSC	JI	ACC	PRE	Params
	**FCN**	50.12 ± 0.12	34.66 ± 0.32	34.43 ± 0.38	34.50 ± 0.47	40.2M
	**SegNet**	77.06 ± 0.55	62.47 ± 0.73	83.39 ± 0.65	69.13 ± 1.40	81.7M
†	**U-Net**	79.06 ± 0.43	65.64 ± 0.74	85.43 ± 0.23	71.95 ± 0.77	190.3M
	**UNet++**	73.52 ± 0.60	58.55 ± 0.71	79.31 ± 1.47	63.05 ± 2.28	39.4M
	**MultiResUNet**	77.00 ± 0.56	63.40 ± 0.52	83.33 ± 0.85	70.95 ± 1.79	59.1M
	**UNet3+**	76.61 ± 1.09	62.61 ± 1.30	82.32 ± 0.74	66.96 ± 1.87	146.9M
	**LinkNet**	78.25 ± 0.50	64.79 ± 0.66	84.65 ± 0.35	70.99 ± 1.08	163.0M
	**FPN**	77.93 ± 0.38	64.32 ± 0.56	84.30 ± 0.64	70.44 ± 1.75	140.9M
	**U2net**	80.38 ± 0.53	67.74 ± 0.67	86.66 ± 0.63	74.87 ± 3.56	216.4M
	**FTL_U-Net**	76.39 ± 0.59	62.32 ± 0.67	82.55 ± 0.21	67.22 ± 0.71	**35.4M**
	**CCBANet**	81.54 ± 0.82	70.76 ± 0.99	84.85 ± 0.48	74.56 ± 1.44	118.0M
◊	**DeepLab-V3**	79.42 ± 0.97	66.48 ± 1.39	85.62 ± 1.27	76.14 ± 2.81	331.5M
	**PSPNet**	79.14 ± 0.57	65.91 ± 0.79	85.27 ± 0.52	72.24 ± 0.49	80.3M
**Ours**	**GCLDNet**	**82.65 ± 0.36**	**70.92 ± 0.53**	**88.27 ± 0.58**	**78.20 ± 1.00**	**59.6M**

†: Encoder–decoder-based methods. ◊: Multi-scale-based methods.The bold values indicate the best results achieved by the corresponding method.

As far as the DSC is concerned, our method is 1.1% higher than the second-ranked method, and about 22.6% higher than the lowest method. Moreover, the other highest evaluation indicators were also obtained by our proposed method. This effect shows that the GCLDNet can fuse the level feature information of deep and shallow gastric cancer lesions, and the feature fusion module adopted the attention mechanism to accurately locate the gastric cancer lesion area. Furthermore, the proposed GCLDNet has the advantage of fewer parameters, only 59.6M, while maintaining high accuracy. This fully proves the effectiveness of our method to detect gastric cancer lesions.

**Figure 7:**
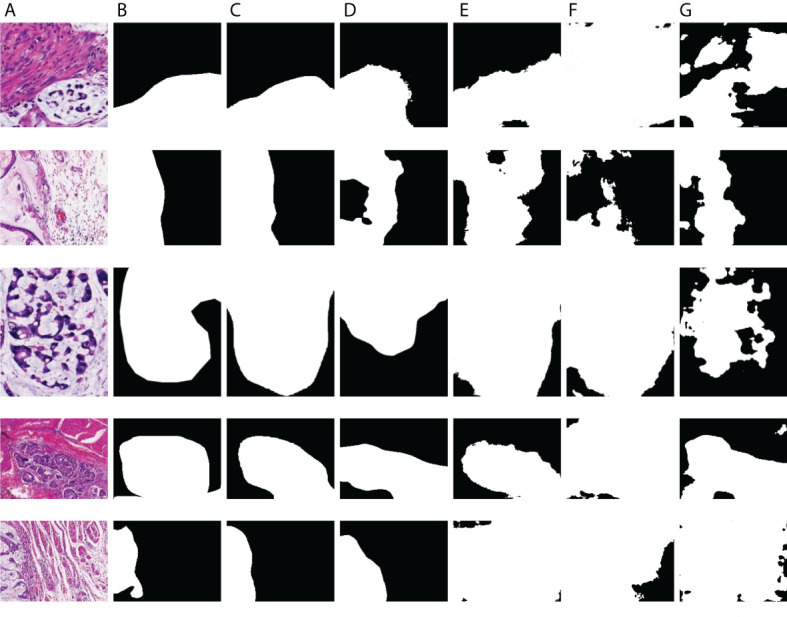
Visualization of the results of gastric cancer dectionon SEED dataset. **(A)** Image; **(B)** Mask; **(C)** GCLDNet; **(D)** PSPNet; **(E)** LinkNet; **(F)** U-Net; **(G)** UNet3+.

To comprehensively assess the superiority of the proposed GCLDNet, the same comparative experiment is also carried out on the BOT dataset. As displayed in **Table 2** and **Figure 7**, the GCLDNet still outperforms other SOTA methods in terms of all evaluation metrics, which once again exhibits the universality and robustness of our method in detecting gastric cancer lesions. In particular, the DSC of our method achieves more than about 1% improvement compared with the second-best method and 7.9% improvement compared with the lowest method. Other experimental conclusions are basically similar to the SEED dataset.

**Table 2:** Segmentation performance of different models on the BOT dataset.

Method type	Methods	DSC	JI	ACC	PRE
	**FCN**	82.04 ± 0.13	60.69 ± 0.28	78.26 ± 0.43	62.61 ± 0.56
	**SegNet**	85.78 ± 0.23	74.55 ± 0.28	50.60 ± 0.31	80.73 ± 0.66
†	**U-Net**	88.76 ± 0.11	79.72 ± 0.44	88.10 ± 0.67	84.51 ± 1.67
	**UNet++**	84.1 ± 0.59	73.30 ± 0.90	83.30 ± 0.55	79.92 ± 0.43
	**MultiResUNet**	87.63 ± 0.20	78.33 ± 0.34	87.56 ± 0.38	84.07 ± 0.86
	**UNet3+**	87.91 ± 0.75	79.02 ± 1.00	87.88 ± 0.60	85.24 ± 1.48
	**LinkNet**	87.83 ± 1.08	78.14 ± 1.73	87.13 ± 1.21	82.71 ± 2.73
	**FPN**	88.58 ± 0.38	79.41 ± 0.87	88.16 ± 0.93	84.95 ± 1.95
	**U2net**	88.12 ± 0.68	79.03 ± 1.03	87.65 ± 0.58	86.38 ± 1.09
	**FTL_U-Net**	86.94 ± 0.51	77.41 ± 0.74	86.13 ± 0.38	81.93 ± 0.71
	**CCBANet**	86.12 ± 0.88	77.26 ± 1.12	89.36 ± 0.71	86.23 ± 0.83
◊	**DeepLab-V3**	87.02 ± 0.21	77.27 ± 0.42	87.43 ± 0.53	86.25 ± 1.05
	**PSPNet**	88.56 ± 0.45	79.08 ± 1.48	88.07 ± 0.82	83.80 ± 2.88
**Ours**	**GCLDNet**	**89.91 ± 0.40**	**81.82 ± 0.62**	**89.49 ± 0.44**	**87.63 ± 1.60**

†: Encoder–Decoder-based methods. ◊: Multi-scale-based methods.The bold values indicate the best results achieved by the corresponding method.

**Figure 8:**
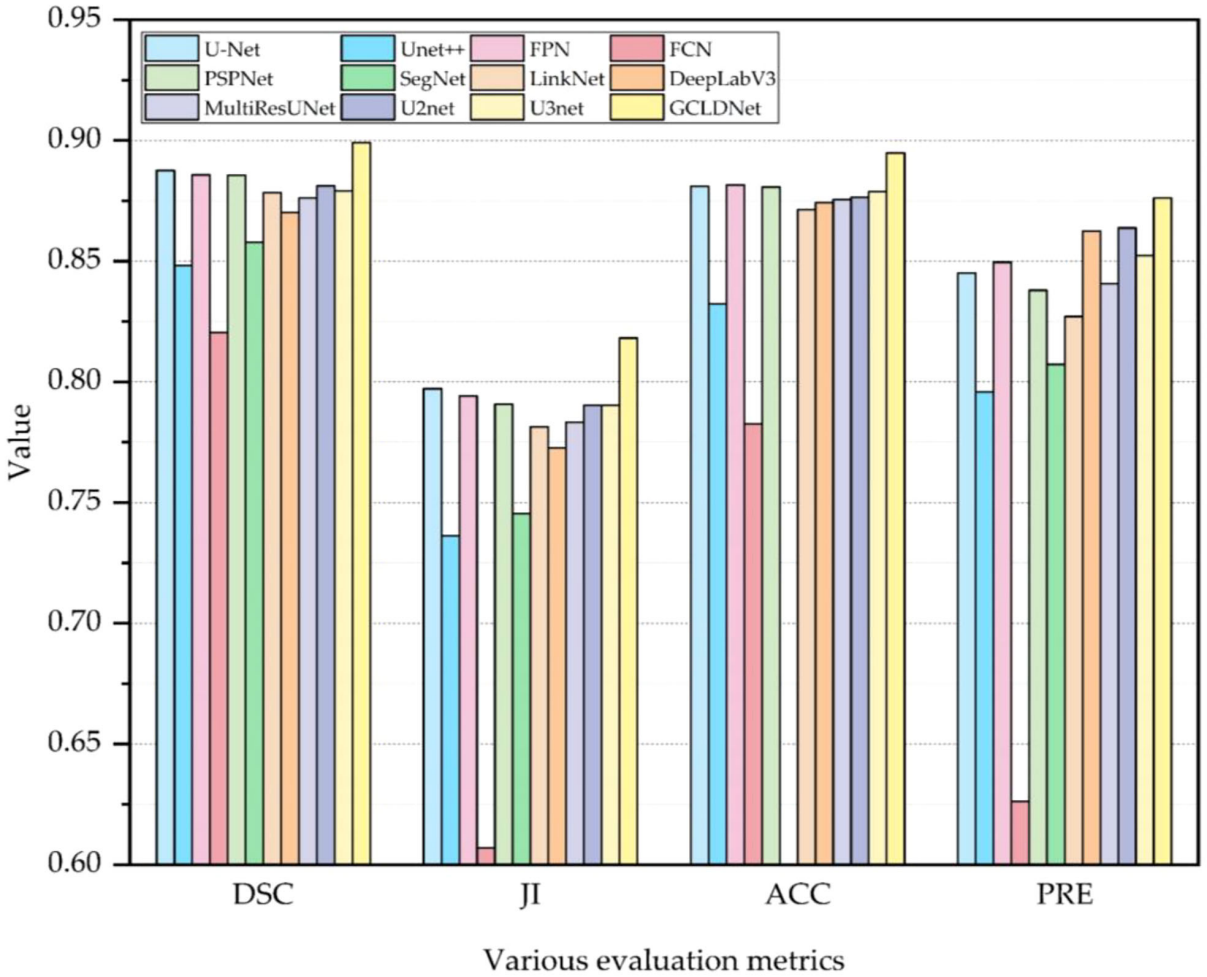
Visual comparison of the performance of different models on BOT Dataset.

**Figure 9:**
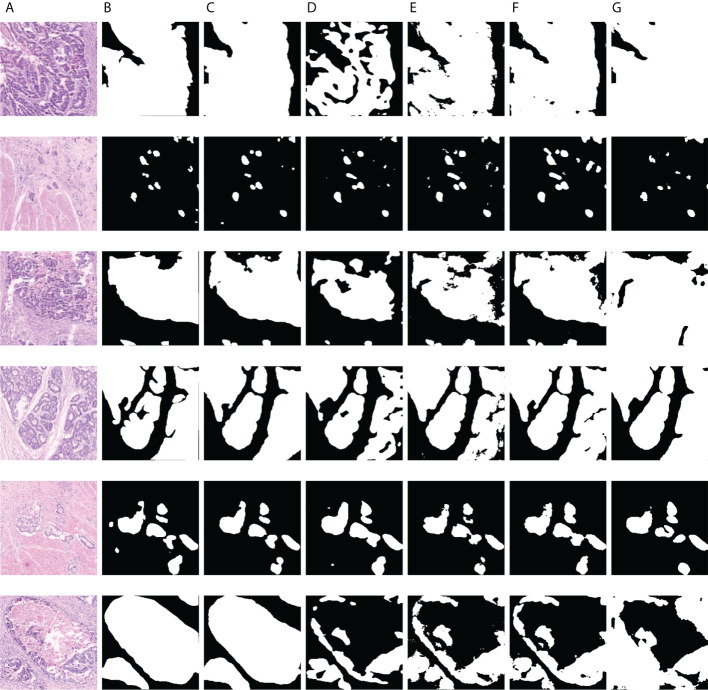
Visualization of the results of gastric cancer detection on the BOT dataset. **(A)** Image; **(B)** mask; **(C)** GCLDNet; **(D)** PSPNet; **(E)** LinkNet; **(F)** U-Net; **(G)** UNet3+.

As displayed in **Figures 8** and **9**, we visualize some representative qualitative segmentation results of the GCLDNet and some other SOTA networks on the SEED dataset and BOT dataset, respectively. These experimental results show that the GCLDNet is more accurate in detecting small lesion areas and more complete in detecting large lesion areas.

### 3.4 Ablation study experiment

To validate the individual contribution of each part of the GCLDNet, ablation experiments are carried out on two datasets of SEED and BOT. The specific method is to divide the GCLDNet into three parts: Baseline, LFA, and AFFM. The specific settings are as follows: (1) The EfficientNet-B4 network (47, 48) is used as Backbone in encoding stage and network and is similar in shape to U-Net, which is called the Baseline method; (2) Baseline+LFA; and (3) Baseline+LFA+AFFM. The experimental results on the SEED dataset are displayed in **Table 3**.

**Table 3:** Ablation study of the GCLDNet method on the SEED dataset.

Methods	DSC	JI	ACC	PRE
**Baseline**	69.13 ± 3.55	50.18 ± 0.35	75.43 ± 2.85	56.75 ± 6.41
**Baseline + LFA**	80.96 ± 0.11	68.44 ± 0.17	88.21 ± 0.27	76.87 ± 2.38
**Baseline + LFA + AFFM**	**82.65 ± 0.36**	**70.92 ± 0.53**	**88.27 ± 0.58**	**78.20 ± 1.00**

The bold values indicate the best results achieved by the corresponding method.

**Table 3** describes that compared with the Baseline method, adding the LFA module can effectively enhance the segmentation accuracy, the Dice coefficient is increased by more than 11%, and other indicators are also greatly improved; the improvement effect is also obvious. This illustrates that the LFA module helps aggregate the deep features of image between different levels and achieve higher-precision segmentation. After continuing to add the AFFM, the Dice coefficient has increased by about 1.6%, and other indicators have also improved. This phenomenon suggests that the AFFM can effectively locate gastric cancer lesion areas through attention mechanism and integrate features of different depths, which can better improve the segmentation performance.

To make the results of the ablation experiment more convincing, the BOT dataset is selected for another ablation experiment. The method and settings of the experiment are consistent with the SEED dataset. The experimental results on the BOT dataset are presented in **Table 4**. From **Table 4**, compared to the Baseline method, the addition of the LFA module once again proves that the segmentation accuracy can be effectively improved, the Dice coefficient is increased by more than 7%, and other indicators have also been greatly improved; the improvement effect is also very obvious. This once again proves that the LFA module can effectively aggregate deep features between different levels. When the AFFM continues to be added, the Dice coefficient increases by about 2.6%. The effectiveness of the AFFM is mainly because of the appropriate utilization of the attention mechanism and the fusion of output features from different depths.

**Table 4:** Ablation study of the GCLDNet method on the BOT dataset.

Methods	DSC	JI	ACC	PRE
**Baseline**	80.11 ± 1.17	67.04 ± 1.54	78.63 ± 1.75	75.25 ± 3.16
**Baseline + LFA**	87.28 ± 0.32	77.38 ± 0.66	86.43 ± 0.22	82.50 ± 0.38
**Baseline + LFA + AFFM**	**89.91 ± 0.40**	**81.82 ± 0.62**	**89.49 ± 0.44**	**87.63 ± 1.60**

The bold values indicate the best results achieved by the corresponding method.

### 3.5 The effectiveness of loss function

**Figure 10:**
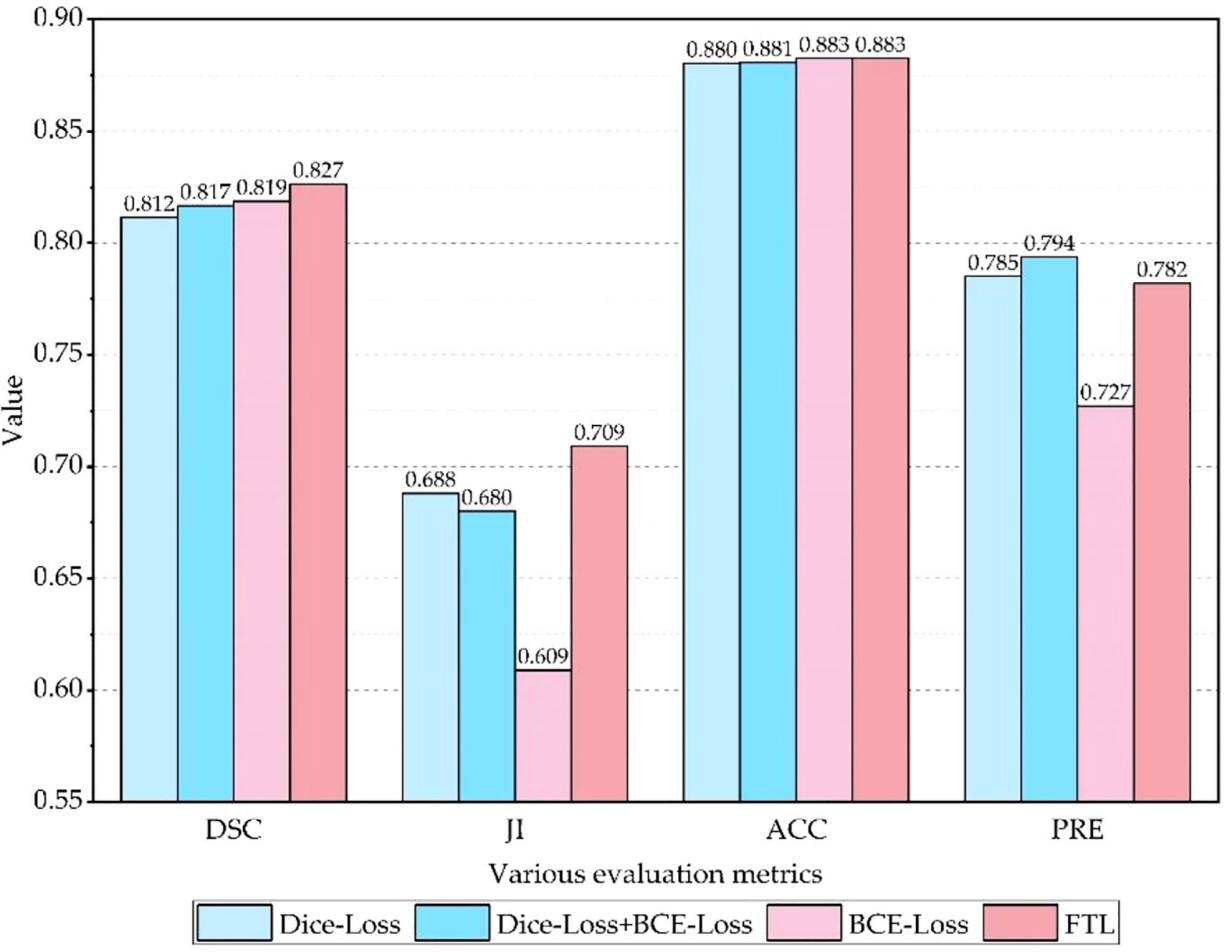
Comparison of loss function performance.

To evaluate the effectiveness of the FTL, comparative experiments are carried out on the SEED dataset, and experimental results are shown in **Table 5** and **Figure 10**. It is clear that the Dice coefficient obtained by using the FTL is the highest, followed by BCE-Loss. FTL is about 0.8% higher than BCE-Loss. Dice-Loss has the lowest Dice coefficient. At the same time, BCE-Loss and Dice-Loss are jointly used as the loss function, and the Dice coefficient is between BCE-Loss and Dice-Loss. These indicate that the FTL can reduce the prediction of false-negative samples, mine difficult samples, and improve the detection accuracy of gastric cancer lesions.

**Table 5:** Effectiveness experiment of FTL loss function.

Loss function	DSC	JI	ACC	PRE
**Dice-Loss**	81.15 ± 0.61	68.81 ± 0.86	88.04 ± 0.90	78.51 ± 2.26
**Dice-Loss + BCE-Loss**	81.68 ± 0.61	68.01 ± 1.44	88.08 ± 0.73	**79.38 ± 1.03**
**BCE-Loss**	81.87 ± 0.52	60.90 ± 1.90	88.26 ± 1.18	72.71 ± 1.23
**FTL**	**82.65 ± 0.36**	**70.92 ± 0.53**	**88.27 ± 0.58**	**78.20 ± 1.00**

The bold values indicate the best results achieved by the corresponding method.

## 4 Conclusions

In this paper, a gastric cancer lesion detection network (GCLDNet) is proposed for the automatic and accurate segmentation of gastric cancer lesions from HIs. At first, the GCLDNet explores an LFA structure in the decoding stage by adding multiple skip connections. The LFA aggregates rich feature information and can make full use of the deep and shallow feature information of GCHIs. Meanwhile, an AFFM is proposed to accurately locate gastric cancer lesions. The AFFM merges attention feature information of different scales, and obtains rich discriminative feature information focusing on gastric cancer lesion areas. Finally, FTL is employed as a loss function to reduce false-negative predictions and mine difficult samples. Experimental results on two GCHI datasets of SEED and BOT show that DSCs of GCLDNet are 0.8265 and 0.8991, ACCs are 0.8827 and 0.8949, JIs are 0.7092 and 0.8182, and PREs are 0.7820 and 0.8763, respectively. Experiments demonstrate that the GCLDNet obtains the best lesion detection effect compared to some SOTA methods. In future research, we will explore the practical application of the model, and use or improve this method for the detection of other cancers.

## Data availability statement

Publicly available datasets were analyzed in this study. This data can be found here: https://www.jseedata.com, https://data.mendeley.com/datasets/thgf23xgy7.

## Author contributions

Conceptualization: XS, LW, and HH. Methodology: XS and LW. Histopathological images re-annotation: YL, JW, and XS. Software: XS and LW. Validation: XS and LW. Investigation: XS and LW. Discussion: XS, LW, YL, JW, and HH. Writing—original draft preparation: XS. Writing—review and editing: XS, LW, YL, JW, and HH. Visualization: XS. Supervision: HH. Project administration: HH. All authors have read and agreed to the published version of the manuscript.

## Funding

This research was supported by the National Science Foundation of China under Grant 42071302 and the Basic and Frontier Research Programmes of Chongqing under Grantcstc2018jcyjAX0093.

## Acknowledgments

Thanks to the publishers of the SEED and BOT datasets.

## Conflict of interest

The authors declare that the research was conducted in the absence of any commercial or financial relationships that could be construed as a potential conflict of interest.

## Publisher’s note

All claims expressed in this article are solely those of the authors and do not necessarily represent those of their affiliated organizations, or those of the publisher, the editors and the reviewers. Any product that may be evaluated in this article, or claim that may be made by its manufacturer, is not guaranteed or endorsed by the publisher.
